# Selective Binding, Self-Assembly and Nanopatterning of the Creutz-Taube Ion on Surfaces

**DOI:** 10.3390/ijms10020533

**Published:** 2009-02-12

**Authors:** Yuliang Wang, Marya Lieberman, Qingling Hang, Gary Bernstein

**Affiliations:** 1 Department of Chemistry, University of Notre Dame, Notre Dame, Indiana 46556, USA; E-Mail: mlieberm@nd.edu; 2 Department of Electric Engineering, University of Notre Dame, Notre Dame, Indiana 46556, USA; E-Mails: qhang@nd.edu (Q.H.); gbernste@nd.edu (G.B.)

**Keywords:** Creutz-Taube ions, Surface, Hydrophilic, Hydrophobic, XPS, AFM, Self-assembled Monolayers, E-beam lithography, PMMA, Nanopatterning

## Abstract

The surface attachment properties of the Creutz-Taube ion, i.e., [(NH_3_)_5_Ru(pyrazine)Ru(NH_3_)_5_]^5+^, on both hydrophilic and hydrophobic types of surfaces were investigated using X-ray photoelectron spectroscopy (XPS). The results indicated that the Creutz-Taube ions only bound to hydrophilic surfaces, such as SiO_2_ and –OH terminated organic SAMs on gold substrates. No attachment of the ions on hydrophobic surfaces such as –CH_3_ terminated organic SAMs and poly(methylmethacrylate) (PMMA) thin films covered gold or SiO_2_ substrates was observed. Further ellipsometric, atomic force microscopy (AFM) and time-dependent XPS studies suggested that the attached cations could form an inorganic analog of the self-assembled monolayer on SiO_2_ substrate with a “lying-down” orientation. The strong electrostatic interaction between the highly charged cations and the anionic SiO_2_ surface was believed to account for these observations. Based on its selective binding property, patterning of wide (∼200 nm) and narrow (∼35 nm) lines of the Creutz-Taube ions on SiO_2_ surface were demonstrated through PMMA electron resist masks written by electron beam lithography (EBL).

## 1. Introduction

The preparation of organized monolayer and multilayer molecular assemblies on solid substrates has been an area of great interest for many years due to their important roles in various applications such as catalysis [1], electrochemistry [2–5], microelectronics [6–8], and especially, in the recently up-and-coming areas of nanotechnology [9] and biotechnology [10]. Various techniques can be used to place molecules on surfaces, such as Langmuir-Blodgett deposition [11,12], spin-coating [13,14], and vapor deposition [15,16]. Compared with these techniques, the self-assembled monolayer (SAM) technique developed by Whitesides, Ulman and Nuzzo [17–19] offers great advantages in that the monolayer films are normally formed through adsorption from solution and are chemically bound to substrates so that reproducible and stable films can be easily obtained. By choosing different types of functional head and tail groups for the SAM molecules, the interaction between these end groups and the substrates could be adjusted to be highly specific and often involve the formation of strong covalent bonds (e.g., thiols on gold, siloxanes on SiO_2_, isocyanates on platinum, carboxylates on alumina, vinyl groups on hydrogen terminated silicon, etc) [17]. With the deposition of SAMs containing different functional groups on the substrates, the surface chemistry of solid substrates could be modified accordingly. Although most of the previous studies on SAMs are related to the formation of organic monolayers that are covalently-bonded to the substrates, there are also a number of studies dealing with the SAMs that were formed through electrostatic or ionic interactions between the charged adsorbates and the substrates [20–32], i.e., an inorganic analog of the self-assembled organic monolayers. With the selective binding property between oppositely charged adsorbates and substrates and also the possibility of forming hydrogen bond between the electron donor groups and OH or NH_2_ groups, molecular patterning [33–41], bioreorganization [42–47] and sequential layer-by-layer deposition [48–50] have been demonstrated in a number of previous studies on various types of inorganic SAMs.

For a recently proposed and developed novel computational architecture, quantum-dot cellular automata (QCA) [51–54], the well-known mixed-valence Creutz-Taube ion [55–58], i.e., [(NH_3_)_5_Ru(pyrazine)Ru(NH_3_)_5_]^5+^, has been selected as a prospective candidate molecule for the implementation of QCA at a molecular scale [59–61]. In the QCA paradigm, the candidate molecules will have to be patterned into arrayed structures to carry out logical computing [51–54]. To achieve this, a proper substrate is necessary to put the molecules on and then to pattern them. Here we are interested in knowing whether the Creutz-Taube ions have the required selective binding properties (for surface patterning) on different types of surfaces, such as hydrophilic versus hydrophobic; and if they do bind, whether they can form a self-assembled inorganic monolayer on the substrate.

Some studies have been done to investigate the surface attachment properties of several ruthenium compounds on modified surfaces where different anchoring groups were used [62–66]. For example, Isied and coworkers have demonstrated the binding of *trans*-[Ru(II)(NH_3_)_4_(H_2_O)_2_] to terminal pyridine or imidazole groups on self-assembled monolayers [67]. In many cases, it has been found that the organic SAM can confer control over the orientation of the adsorbed inorganic molecules, which argues for strong interactions between the SAM surface and the inorganic molecules layer.

In this study, two types of surfaces, i.e., –OH group terminated hydrophilic surfaces and –CH_3_ group terminated hydrophobic surfaces, were used to study the selective binding properties of the Creutz-Taube ions. X-ray photoelectron spectroscopy (XPS) was the main technique used in this study to detect the binding of the molecules on surfaces. XPS can easily detect an impurity atom on a surface, even when the surface coverage is only about one in one hundred substrate atoms. With this sensitivity the selective binding of the Creutz-Taube ions on surface could easily be observed. Silicon [100] wafers covered with a freshly grown native oxide were used as the hydrophilic substrate for our initial binding study. The existence of silanol groups (Si-OH) on silica surfaces was first postulated by Hofman in 1934 [68]. Various analytical techniques have allowed silica scientists later to confirm and expand the view of the silica surface in terms of silanol groups, siloxane bridges, and hydrogen-bonded water [68]. It is now generally accepted that surface silicon atoms tend to have a complete tetrahedral configuration and that in an aqueous medium their free valence becomes saturated with hydroxyl groups and form silanol groups with a density of SiOH on SiO_2_ ∼ 4.6 −OH/nm^2^ [68]. Besides SiO_2_, another way to prepare a hydrophilic surface is to grow SAMs terminated with –OH or some other hydrophilic functional groups such as NH_2_ and –PO_3_H_2_ onto the substrates. Here –OH terminated hydroxyundecanethiol (HUT) was used in this study to form a hydrophilic monolayer on gold substrate. Similarly, –CH_3_ terminated octadecyltrichlorosilane (OTS) and octadecanethiol (ODT) were used to form hydrophobic surfaces on SiO_2_ and gold substrates respectively. For surface patterning purpose, the binding of the Creutz-Taube ions on another hydrophobic surface – poly(methyl methacrylate) (PMMA) covered SiO_2_, was investigated as well. Besides XPS, ellipsometry measurement and atomic force microscopy (AFM) were also used to track monolayer formation and characterize the adsorbed species. Our results indicated that the Creutz-Taube ions only bound to hydrophilic surfaces, such as SiO_2_ and –OH terminated organic SAMs, but not to hydrophobic surfaces, such as OTS and ODT SAMs or PMMA thin film. The counter ions of the Creutz-Taube ions, either toluenesulfonate (TOS) or hexfluorophosphate (PF_6_) as used in this study, were not observed on any of the two types of surfaces. Additional experimental results also suggested that the attached Creutz-Taube ions could form a self-limiting monolayer on SiO_2_ with a “lying-down” orientation. Because of its selective-binding property, patterning of wide (∼200 nm) and narrow (∼35 nm) lines of the Creutz-Taube ions on SiO_2_ surface were demonstrated through PMMA masks written by electron beam lithography (EBL).

## 2. Experimental Section

### 2.1. Chemicals and Materials

The Creutz-Taube ions used in this study were prepared using published procedures and were precipitated and thus collected with either *p*-toluenesulfonate (TOS) or hexafluorophosphate (PF_6_) as their counter ions [55–58]. Octadecyltrichlorosilane (OTS), octadecanethiol (ODT), 11-mercapto undecanol (HUT) were received from Aldrich and used without further purification. P-doped single-sided polished Si wafers (100 orientation) were obtained from MEMC Electronic Materials, Inc. Gold wire (99.99%) was obtained from Aldrich, and mica sheet was from Woodman Associates, Inc. Other solvents were used directly as received.

### 2.2. Substrate Preparation

The preparation of Si substrates covered with freshly grown native oxide or ultrasmooth complete OTS monolayer followed a previously described procedure [69]. Gold substrates were prepared by evaporating ∼ 800 Å of gold onto freshly cleaved mica sheets using a Ladd evaporator (Ladd Research Industries Inc.) under a vacuum of 10^−6^ Torr. The gold evaporation rate was kept at 0.6 Å/s. The surfaces were annealed in a hydrogen flame immediately before immersion in derivatization solutions. This annealing step cleans the surface and allows epitaxial reconstruction of the Au to form large terraces of Au [111]. Self-assembled monolayers of alkanethiols on gold with terminal –CH_3_ and –OH functional groups were prepared by soaking gold substrates (cut to 1 cm × 1 cm) in 1 mM solution of ODT or HUT in chloroform overnight. Upon removal from the solution, the samples were washed thoroughly with chloroform and then blown dry with nitrogen. The HUT covered gold substrate showed contact angles under 20° and an ellipsometric thickness of 13.8 ± 0.4 Å confirming the self-assembled monolayer formation on the substrate. For ODT covered gold substrate, the value of 115±5° was obtained in the contact angle measurement indicating the expected hydrophobicity of the surface.

### 2.3. Surface Attachment of the Creutz-Taube ions on substrates

A concentration of 0.5 mM Creutz-Taube ion in DI water was used. In typical preparations, the substrates were soaked in the solution for three hours in a dark environment. Running DI water was used for final rinsing before the samples were blown dry with a strong flow of nitrogen.

### 2.4. Electron Beam Lithography Patterning of the Creutz-Taube Ions

Oxidized Si wafers (∼100 nm thick SiO_2_ on an n-type silicon formed by dry oxidation at 1200°C for one hour) were cleaned prior to spinning coating of PMMA. First, the oxidized wafers were ultrasonicated in trichloroethylene and acetone for 10 min each, rinsed in DI water; soaked in a “RCA1” bath at 75 °C for 15 min, then soaked in a “RCA2” bath at 75 °C for 15 min, and finally soaked in 1:10 HF solution for 10 s and rinsed in DI water. The wafers were baked at 180 °C for 5 min on a hot plate to drive off moisture from the surface. The resulting surface oxide was estimated to be about 90-nm-thick. A 30-nm-thick layer of 950 kDa PMMA was spun on and baked at 180 °C for 3 min. Finally, high-resolution patterns were exposed in our customized EBL system (modified from an Amray 1400 SEM) at a beam current of 10 pA and acceleration voltage of 50 keV. After EBL exposure, the wafer was soaked in developer [1:3 methyl isobutyl ketone: 2-propanol (IPA) with 1.5% methyl ethyl ketone] for 30 s, and then rinsed with IPA, resulting in trenches in the PMMA film exposing portions of the SiO_2_ surface. After drying with N_2_, the wafer was soaked in 5 mM aqueous solution of the Creutz-Taube ions for 3 h, then rinsed with DI water and blown dry. Acetone or dichloromethane (DCM) was used to remove the unexposed PMMA.

### 2.5. XPS measurement

X-ray photoelectron spectroscopy (XPS) was done using a Kratos XSAM 800 with an Al Kα X-ray source (1486.6 eV). The takeoff angle was fixed at 90°. The samples were mounted on sample stubs with conductive carbon tape. All peaks were fitted with Gauss-Lorentz peaks using the Kratos Vision II software to obtain peak area information. A linear base line was used in the fitting processes.

### 2.6. Ellipsometric Measurement

The thicknesses of the Creutz-Taube ions on SiO_2_ substrate were obtained using an Rudolph AutoEl III ellipsometer. First, the thickness of the thin native oxide layer on a cleaned native SiO_2_ substrate was measured. Then after soaking the substrate in 5 mM solution of the Creutz-Taube ion for more than three hours, the sample was rinsed with copious running water and blown dry with a strong flow of nitrogen. The measurement was performed using 632.8 nm He/Ne laser light incident upon the sample at 70°. Both a single-layer and double-layer models were used to verify the validity of the thickness measurement. For each sample, the measurement was done at several places on the surface and the results were averaged. A refractive index of 1.46 was assumed for the SiO_2_ surface [68] and 1.50 for the Creutz-Taube SAMs on SiO_2_ surface [17].

### 2.7. Contact Angle Measurement

All measurements of contact angles are advancing angles and were performed with a KRUSS G 10 contact angle instrument. On each sample at least four different locations were measured and results were averaged.

### 2.8. AFM Measurement

AFM was carried out using a Digital Instruments (Santa Barbara, CA) Multimode Nanoscope III instrument operating in tapping mode. The tips used were tapping mode etched silicon probes (Olympus) with a tip radius of 15–35 nm. The imaging setpoint was set for 1.5 V. Image analysis was performed offline using roughness and section commands provided in the AFM software.

## 3. Results and Discussion

### 3.1. XPS Measurements on Bare Substrates and Bulk Powder Samples as Control Experiments

**Figure 1.**
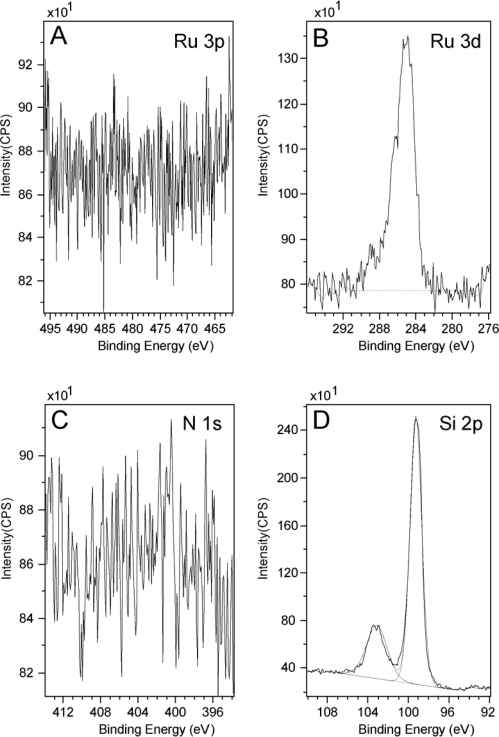
XPS spectra of a bare SiO_2_ substrate before soaking in the Creutz-Taube ion solution. Shown in (A), (B), (C), and (D) are the XPS regions corresponding to element components of Ru 3p, Ru 3d, N 1s and Si 2p, respectively.

Before we carried out the surface attachment studies on the Creutz-Taube ions, as a control experiment, all of the bare substrates (both hydrophilic and hydrophobic) were first checked by XPS to ensure there was no N, Ru, or S on the surface. As a typical example, Figure 1 gives the result of an XPS measurement for Ru 3p, Ru 3d, N 1s and Si 2p region on a bare SiO_2_ substrate, from which we may clearly see that no nitrogen or ruthenium was observed. Here all peaks were fitted with Gauss-Lorentz peaks using the Kratos Vision II software to obtain peak area information. A linear base line was used in the peak fitting processes.

**Figure 2.**
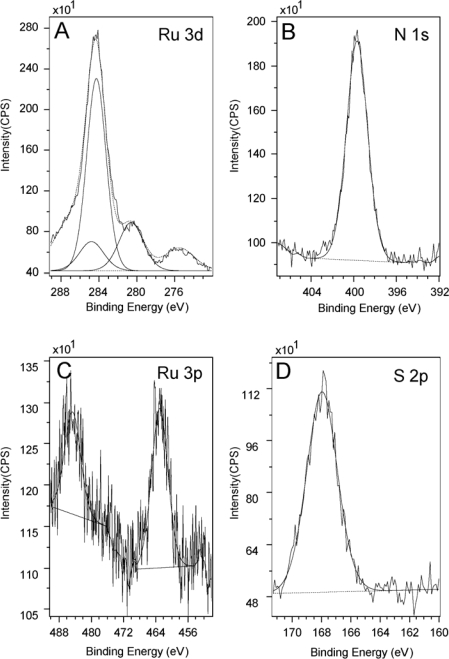
XPS spectra of a powder sample of the Creutz-Taube ions, with (TOS)^−^ as the counter ions. Shown in (A), (B), (C), and (D) are the XPS regions corresponding to element components of Ru 3d, N 1s, Ru 3p and S 2p, respectively.

The peak centered at 284.5 eV, as shown in the Ru 3d region (Figure 1B), is in fact the C 1s peak originating from a large amount of adventitious carbon. For Ru 3d, we would expect two peaks in the region of 284 eV ∼ 286 eV and 280 eV ∼ 282 eV which correspond to spin 3d^3/2^ and 3d^5/3^ respectively [70–73]. Here we see no peak corresponding to 3d^5/3^ in the latter region in Figure 1B. In another control experiment, a powder sample of the Creutz-Taube ion with toluenesulfonate (TOS) as its counter ion was also prepared and measured using XPS. Figure 2 gives the corresponding results for a survey scan and high resolution scans at specific regions. The peaks of Ru (3p, 3d) and N (1s) from the Creutz-Taube cations and S (2p) from the anion are seen in this figure, as expected.

Due to the limited conductivity associated with the sample, charge accumulation caused about a 2 eV binding energy shift. The conventional method of calibration using the C 1s peak of adventitious carbon at 284.5 eV was used for the powder sample. For Ru 3d, because of its overlapping with the intense C 1s peak, only the 3d^5/2^ peak is observed at 280.5 eV, the Ru 3d^3/2^ component is buried under the C 1s peak at 284.5 eV and is not clearly resolved in our spectra. Here it was fitted as a peak about 69% area of that of the Ru 3d^5/2^ component and with a spin orbit splitting of 4.1 eV [70–73]. For Ru 3p, two peaks with the binding energy of 461.8 eV and 484.7 eV corresponding to spin 3/2 and 1/2 are observed. The N 1s peak appears at 399.7 eV as shown in Figure 2B. In the Creutz-Taube ion, there are two nitrogen sources, one is from the NH_3_ groups and the other one is from the pyrazine group. Since both types of nitrogen are connected to the ruthenium atoms, and in addition because of the resolution of the spectra, the binding energy difference for these different sources of nitrogen is also not obvious. The sulfur 2p peak from the TOS counter ions is observed at 168 eV, which is consistent with the S(IV) oxidation state. We noticed that as a well-known mixed-valence complex, XPS studies on the Creutz-Taube ions had been done a few times previously [70]; we found here that our observations on the peak positions of the binding energy agree well with the reported literature results.

From XPS measurement we can also obtain atomic concentrations of each element ***C_i_*** in the molecules using eq 1, where ***A_i_*** is the peak area of element ***i*** and ***S_i_*** is the sensitivity factor of element ***i***.

(1) $$C_{i}=\frac{A_{i}/S_{i}}{\sum_{i}\left(A_{i}/S_{t}\right)}\times 100$$

Table 1 lists the peak positions of the binding energy and atomic concentrations for N, Ru and S obtained from this powder sample. We can see that the observed N to Ru ratio is 6.12 ± 0.17, which is in good agreement with the theoretical ratio of 6:1 for this complex. For the ratio of S to Ru, the observed value of 2.71± 0.19 is also close to the theoretical expectation of 2.5.

### 3.2. Selective Binding Properties on Surfaces

**Figure 3.**
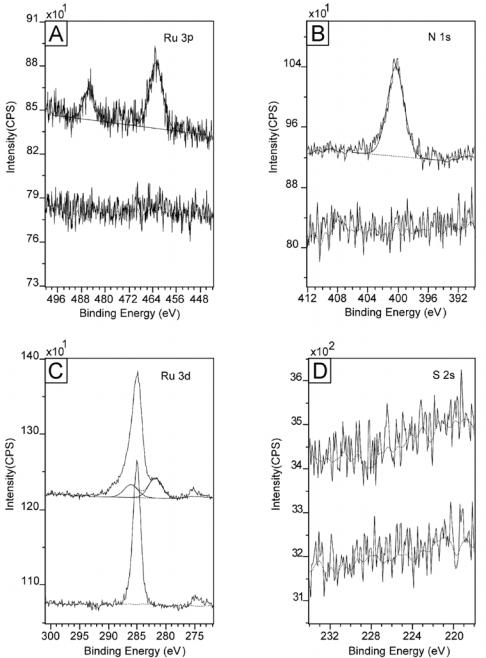
XPS spectra of a SiO_2_ substrate (top curves) and an OTS covered SiO_2_ substrate (bottom curves) after soaking in an aqueous solution of Creutz-Taube ions. Shown in (A), (B), (C), and (D) are the XPS regions corresponding to element components Ru 3p, N 1s, Ru 3d and S 2s, respectively. All peaks were fitted with Gauss-Lorentz peaks to obtain peak area information. A linear base line was used in the peak fitting processes.

The selective binding properties of the Creutz-Taube ion were investigated on several hydrophilic and hydrophobic substrates. The first hydrophilic substrates we tested were silicon [100] wafers covered with a layer of freshly grown native oxide of about 10 Å thickness; and the same types of silicon wafers but covered with a complete OTS monolayer were used as the hydrophobic substrates. After the substrates were immersed in 5 mM aqueous solutions of the Creutz-Taube ions for three hours, they were taken out from the solution and then rinsed with running water for at least 5 min before finally drying in a stream of nitrogen. Figure 3 shows the obtained XPS results for both the hydrophilic silicon native oxide substrate (top lines) and the hydrophobic OTS covered silicon substrate (bottom lines) after exposure to the Creutz-Taube ions. High resolution scans were performed to determine the binding energies and atomic concentrations of Ru and N. From this figure, we see that only on the hydrophilic SiO_2_ surface where both Ru and N were observed. In contrast, neither of them was detected on the OTS-covered SiO_2_ substrate.

For convenience, here the Si 2p peak (99.3 eV) was used to calibrate the binding energy shift caused by surface charging effects. Due to the different calibration methods used here and in the powder sample (Figure 2), the C 1s peak of the adventitious carbon now appears at 285.4 eV, which is upshifted about 1 eV. When this factor is taken into account, after the Creutz-Taube ion attached to SiO_2_ substrate, the binding energy of Ru (485.7 eV, 462.4 eV for 3p and 281.5 eV for 3d) and N (400.0 eV, 1s) peaks are close to those obtained for the powder sample, implying that the molecules did not undergo obvious chemical changes after binding to the SiO_2_ surface. Based on the peak area, the observed ratio of N (1s) to Ru (3p) is about 5.92, which is consistent with the expected ratio of 6:1 if (NH_3_)_5_Ru-pz-Ru(NH_3_)_5_ core of the molecule remains intact after attaching to the surface. For the operation of QCA, it is crucial to keep the intervalence-charge-transfer property of the Creutz-Taube ion on surfaces. If the molecules break on the surface, this charge-transfer process between the two Ru atoms would not be possible. The counter ions of the molecules were also investigated and the result is shown in Figure 3D. Because of the existence of a plasma band for SiO_2_ at a region that can overlap with the S(IV) 2p peak, S 2s was measured instead to check the binding of the counter ions on the substrate. Here we see no signal of sulfur, indicating no binding of TOS.

**Figure 4.**
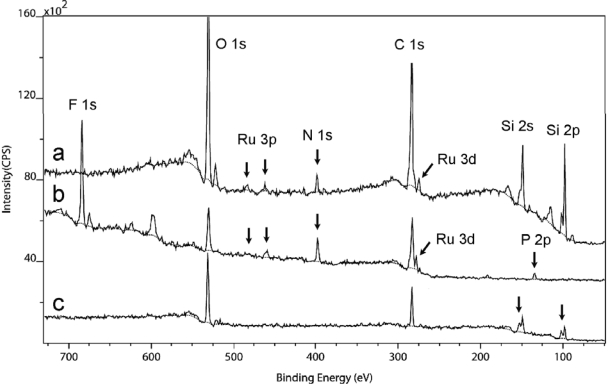
XPS spectra of a SiO_2_ substrate (top curve) and an OTS covered SiO_2_ substrate (bottom curve) after soaking in an aqueous solution of Creutz-Taube ions. The XPS spectrum of a powder sample of Creutz-Taube ions, with (PF_6_)^−^ as the counter ions, is also shown in the figure (middle curve).

In order to find out whether the lack of anion binding is true for other counter ions, the Creutz-Taube ions with a different counter ion – (PF_6_)^−^ – was prepared and used to study its selective binding on the same SiO_2_ and OTS-covered SiO_2_ substrates. The result is shown in Figure 4. Here XPS spectra obtained from survey scans are used and compared. The result of the XPS measurement on a corresponding powder sample was also shown in the middle of this figure. The F 1s peak corresponding to the (PF_6_)^−^ anion in the powder sample is indicated in this figure. Here we see again the appearance of both Ru and N on the SiO_2_ surface but not on the OTS covered SiO_2_ surface. For both SiO_2_ surface and the OTS-covered SiO_2_ surface, both the F 1s peak and the P 2p peak observed in the powder spectra were not observed even though F 1s has a very high sensitivity factor for XPS detection [74]. So from both of these two tests, we can conclude that the Creutz-Taube ions only bound to the SiO_2_ substrate, but not to the OTS-covered SiO_2_ substrate. The two counter ions – TOS and PF_6_ as used in this study, showed no binding on either the hydrophilic or the hydrophobic surfaces.

**Figure 5.**
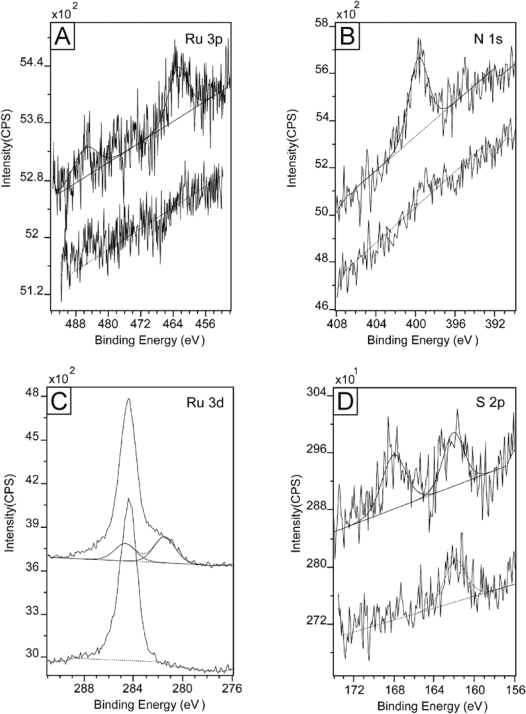
XPS spectra of a HUT covered gold substrate (top curves) and an ODT covered gold substrate (bottom curves) after soaking in an aqueous solution of Creutz-Taube ions. Shown in (A), (B), (C), and (D) are the XPS regions corresponding to Ru 3p, N 1s, Ru 3d and S 2p, respectively.

Another type of surfaces used in the selective binding study were hydrophilic organic SAMs (i.e., HUT) or hydrophobic organic SAMs (i.e., ODT) on gold. The surface attachment samples were prepared the same way as those used in the SiO_2_ and OTS/SiO_2_ binding tests. Figure 5 gives the corresponding XPS results of the binding property on these two substrates. The Au 4f peak at 84 eV was used to calibrate the peak positions of all other elements. From this figure, both Ru (e.g., Ru 3p at 484.9 eV and 462.4 eV) and N (1s, 399.8 eV) are seen on the HUT covered gold substrate, but not on the ODT covered gold substrate, which indicated no binding of the Creutz-Taube ion on this hydrophobic surface. For the HUT covered gold substrate, the binding energy of both Ru and N peaks were similar to the same elemental peaks of the Creutz-Taube ions on SiO_2_ substrate. The intensity, however, appears weaker than that on SiO_2_ as manifested by the signal/noise ratio shown in this figure. The ratio of the atomic concentration of N to Ru (5.74:1) remains close to the value obtained for the powder sample indicating the molecules still appeared to be intact. Since the SAMs has the S-Au bond on the gold substrate, both substrates were expected to observe S peaks on their surfaces. Here peaks centered around 162 eV were assigned to S 2p in the thiol groups [74]. For the HUT covered gold substrate, we also observed an additional S 2p peak with binding energy around 168 eV. By comparing to the S 2p peak from the powder sample (Figure 2D), we can assign this additional peak to the S in the TOS anion. So for the second set of hydrophilic and hydrophobic substrates, the Creutz-Taube ion also selectively bound only on the hydrophilic surfaces, but not on the hydrophobic surface. Different from the binding on the hydrophilic SiO_2_ substrate, here both cations and anions were observed on the HUT covered gold substrate.

**Figure 6.**
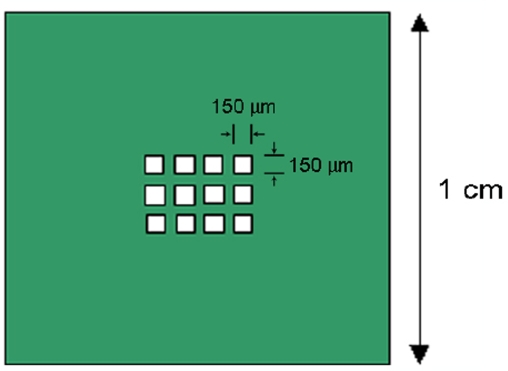
Schematic illustration of the partially exposed SiO_2_ substrate covered by PMMA. The white areas are the twelve 150 × 150 μm open SiO_2_ squares.

**Figure 7.**
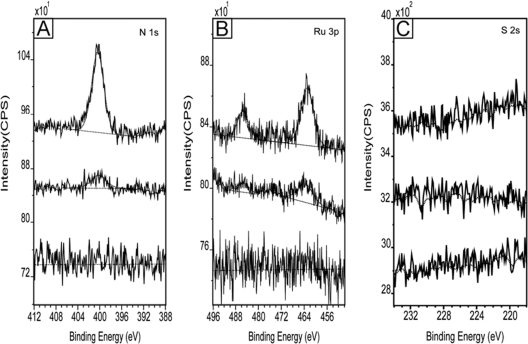
Comparison of the binding of the Creutz-taube ions on three different substrates: a SiO_2_ substrate (top curve), a partially exposed SiO_2_ substrate covered by PMMA (middle curve) and a PMMA fully covered SiO_2_ substrate (bottom curve). Shown in (A), (B) and (C) are the XPS regions corresponding to element components N 1s, Ru 3p and S 2s, respectively.

Since for the implementation of QCA, all candidate molecules will have to be patterned into arrayed structures on a surface in order to carry out logical computing, for the purpose of surface patterning, the selective binding property of the Creutz-Taube ions on PMMA, a widely-used hydrophobic type electron beam resist, was also investigated and compared with that on SiO_2_. In addition, in order to investigate the selective binding property of the Creutz-Taube ions at a micrometer scale, a partially PMMA-covered SiO_2_ substrate with twelve 150×150 μm^2^ open SiO_2_ squares with 50 μm spacing between each square (Figure 6) was also prepared by electron beam lithography. Figure 7 shows the obtained XPS result for a fully PMMA-covered SiO_2_ substrate (bottom), a bare SiO_2_ substrate (top) and a micropatterned SiO_2_ substrate (middle) after they were soaked in the Creutz-Taube ion water solution and then rinsed. The total absence of N and Ru peaks in the bottom lines indicates that the Creutz-Taube ions did not bind to the PMMA surface. For the micropatterned sample, because the XPS system incorporates no mechanism for alignment, the beam was swept until a maximum signal was obtained.

In such a measurement, about ten percent of the area exposed by the XPS beam contained the squares (XPS beam size ∼ 3 mm^2^, total area of open SiO_2_ ∼ 0.27 mm^2^). From Figure 7, we see the peak intensities of Ru 3p and N 1s are reasonably large for the area exposed when compared to the SiO_2_ sample (intensities ratio ∼ 1:10). The measured N:Ru ratio of 5.67 indicates that the Creutz-Taube ion selectively bound to the oxide squares formed by EBL in much the same way as they bound to the large area bare SiO_2_ substrate. Sulfur 2s region was scanned to determine the binding of TOS anions on the PMMA fully- or partially-covered substrates. From Figure 7C, we see none of the substrates showed any sulfur peak, indicating no binding of the TOS on PMMA surface.

As a summary, Table 2 below lists all the XPS peak positions, relative atomic concentrations and the selective binding properties of the Creutz-Taube ion on the hydrophilic and hydrophobic substrates we tested.

From the above experiments, it is clear that the Creutz-Taube ion only binds on hydrophilic surfaces, but not on hydrophobic ones. We believe it is the attractive or repulsive interaction between the Creutz-Taube ions and the surface that lead to such selective binding properties. The isoelectric point of SiO_2_ surface is around 3.8 [68], so at any pH larger than 3.8, the surface of SiO_2_ carries a significant amount of negative charges. Since the Creutz-Taube molecules are highly charged cations, and the pH of the solution is neutral, the strong electrostatic attraction between the cations and the anionic surface will make them bind together [48]. For the same reason, since the counter ions and the surface are both anionic, due to Coulombic repulsion, the binding of the anions, such as PF_6_ or TOS, on SiO_2_ surface is not favorable when compared to the cations binding. It is very likely that the anions will be removed during any solvent rinsing processes.

For the –OH terminated SAMs grown on gold, the surface charge will be smaller than for SiO_2_. Because of their high pKa values (> 10), the –OH groups are not easy to dissociate. Despite the lack of a large negative surface charge, hydrogen bonds can likely still be formed between the H atoms existing in the NH_3_ groups of the cations and the oxygen atoms in the OH groups on the surface. The XPS signal of Ru and N from the Creutz-Taube ions on to the HUT surface is weaker than the signals on the SiO_2_. This is most likely due to the fact that such hydrogen bonding is less strong than the electrostatic interaction between the highly charged cations and the anionic SiO_2_ surface. Since there is no repulsive force between these OH groups on the SAMs and the negatively charged counter ions, it is possible to form hydrogen bonds between the O atoms in TOS and the OH group from the SAM layer. Indeed, the HUT surface is the only one that the counter ions bind.

For hydrophobic surfaces, such as the long chain CH_3_ terminated SAMs on SiO_2_ or gold, the only possible interaction between the Creutz-Taube ion and these CH_3_ functional groups is the Van der Waals force, which is much weaker, compared to hydrogen bonds and electrostatic interactions. During solvent rinsing, both cations and anions could be easily removed from the hydrophobic surfaces.

### 3.3. Inorganic SAMs: Characterization of the Creutz-Taube Ion on SiO_2_

At this point, we know that the Creutz-Taube ions only bind to hydrophilic surfaces, but how they bind to the surface after their attachment, such as the molecular orientations and whether they form monolayer or multilayers on the surfaces, is still unknown. In order to find out the answers to these questions, time-dependent XPS, ellipsometry, and AFM surface roughness analysis and line section analysis were carried out to acquire more details on the attachment of the Creutz-Taube ions on surfaces. For the interests of QCA realization, the following discussion was focused on understanding the binding of the Creutz-Taube ions on SiO_2_ substrate.

When a SiO_2_ substrate is soaked in the Creutz-Taube ion solution, the Creutz-Taube ion in the solution will begin to attach to the adsorption sites on SiO_2_. With increased soaking time, more and more Creutz-Taube ions are expected to attach to the SiO_2_ surface, so the surface coverage or the Ru to Si ratio in the XPS measurement will keep increasing with the increasing sample soaking time in solution. If the attachment of the Creutz-Taube ion on SiO_2_ surface is due to the strong electrostatic interaction between these highly charged cations and the deprotonated surface –OH groups, once all the available binding sites are occupied, the attachment of the Creutz-Taube ion on SiO_2_ surface should stop. It is possible that the Creutz-Taube ion will keep adsorbing onto the already formed the Creutz-Taube ion layer, but the interaction between these species will be very weak when compared to the strong interaction between the first layer of the Creutz-Taube ion and the negatively charged surface. During the rinsing of the sample, these weakly bound Creutz-Taube ion should be easily removed. So it is expected that for the binding of the Creutz-Taube ion on SiO_2_ surface, there should be a self-limiting process to form a monolayer of the Creutz-Taube ion on the SiO_2_ surface, so the Ru to Si ratio would reach a constant value after a certain time of soaking the SiO_2_ substrate in the Creutz-Taube ion solution.

**Figure 8.**
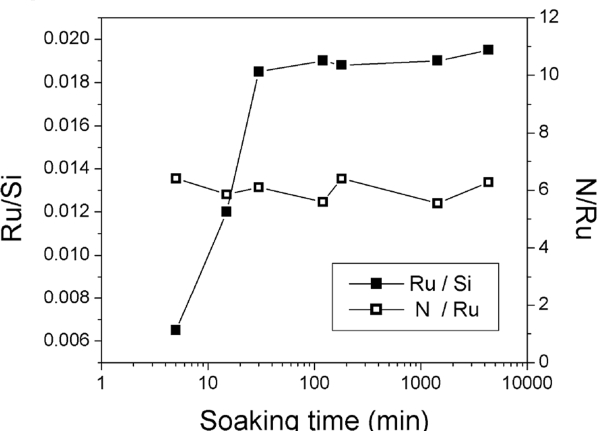
Time-dependent XPS results of the attachment of the Creutz-Taube ions on SiO_2_ substrate. The N/Ru and Ru/Si ratios were plotted against the soaking time of the sample substrates.

XPS samples with different soaking time ranging from 5 min to 1 week in the Creutz-Taube ion solution were used. The atomic concentration of the Ru 3p at 462 eV and 484 eV and the total 8 concentration of the Si 2p at 99.3 eV (0) and 103.3 eV (IV) were compared for all the samples. Figure 8 shows the results. Saturation coverage was achieved after about 30 min soaking time. The Ru to Si ratio reached a constant value around 0.02. The corresponding N/Si ratio was also calculated and it gave a value around 0.11. The observation of a saturation coverage indicates a self-limiting adsorption process of the Creutz-Taube ion on SiO_2_ surface. This result is crucial for the fabrication of QCA devices on surface. A multilayer formation of the Creutz-Taube ion would make the separation of these cells difficult and thus almost impossible to pattern them into arrayed single layer structures. Also shown in Figure 8 is the corresponding N/Ru ratio for these samples prepared with different soaking time. Here almost constant values were observed, and the averaged ratio is 5.94±0.59, which is very close to the expected theoretical value of 6. This result further suggested that the Creutz-Taube ions did not undergo dissociation and remain intact on the SiO_2_ surface regardless how long the substrate were soaked.

Though we know the surface density of the Si-OH group is about 4.6 per nm^2^ on SiO_2_, the surface coverage of the Creutz-Taube ions on SiO_2_ can not be obtained directly from the Ru to O or Ru to Si ratio [62]. The reason is that the ratio between Ru and O or Si does not relate only to the surface Si-OH group, the intensity of O or Si XPS peaks always has contributions from the silicon oxide underneath the surface layer considering the typical XPS penetration depth is larger than 50 Å. So the value of Ru to Si (0.02) or N to Si (0.11) ratios could not get the surface area occupied by each of the Creutz-Taube ion, however, by comparing this ratio to other molecules with known coverage (area per molecule) and N to Si value in similar condition, we can obtain this information indirectly. The following shows how we used APTES (NH_2_-(CH_2_)_3_-Si-(OC_2_H_5_)_3_) to calculate the coverage for the Creutz-Taube ion on native silicon oxide.

It has been known that for fully covered self-assembled organic monolayers, the area occupied by each molecule is around 22 Å^2^ [17–19, 68]. APTES which contains one NH_2_ group per molecule, so if we use the same silicon substrate as used in the Creutz-Taube ion surface attachment studies to form APTES SAMs, by comparing the N/Si ratio for these two molecules, we can calculate out the surface coverage for the Creutz-Taube ions on silica using Eq. 2:

(2) $$\text{Area}\;\text{per}\;\text{Creutz}-\text{Taube}\;\text{ion}\;=\frac{{\frac{N}{Si}}_{\mathrm{APTES}}}{{\frac{N}{Si}}_{CT5}}\times 12\times 22\;{\overset{˚}{\text{A}}}^{2}$$

**Figure 9.**
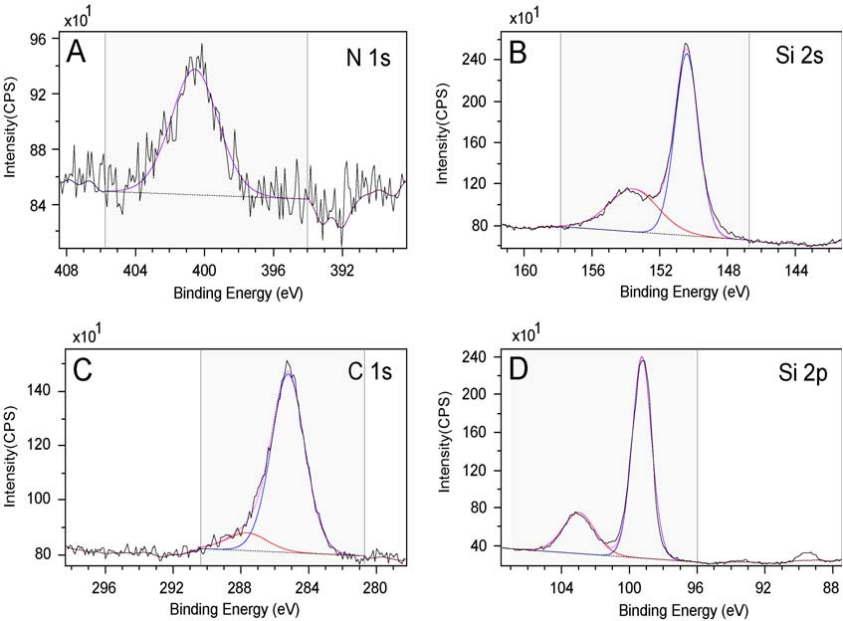
XPS spectra of a APTES monolayer covered SiO_2_ substrate. Shown in (A), (B), (C), and (D) are the XPS regions corresponding to element components of N 1s, Si 2s, C 1s and Si 2p, respectively.

The factor of 12 is due to the fact that each of the Creutz-Taube ion contains 12 nitrogen atoms. The APTES SAMs on native silicon oxide were prepared in a similar way as OTS SAMs on native silicon oxide [69]. Monolayer formation was confirmed by ellipsometric measurement where a thickness of ∼6–7 Å (theoretical value based on CHEM 3D model ∼ 6 Å) was obtained. Figure 9 shows the XPS results for APTES on the same type of SiO_2_ substrate, here the ratio of N to Si (both IV and 0) is 0.0525 for the APTES SAMs. Considering the saturated N to Si ratio (0.11) for the Creutz-Taube ion on silica surface, the calculated area per cation on surface is ∼ 120 Å^2^. For fully covered Creutz-Taube ion monolayer, this value is close to the theoretical value of 85 Å^2^ for the area when the molecule adopts a lying-down orientation. Also because there are about 4.6 negative charges per nm^2^ on SiO_2_ surface, for a fully covered Creutz-Taube ion monolayer, each 5+ charged cation would occupy a surface area ∼ 110 Å^2^ to make it neutralized. This coverage result (120 Å^2^) in fact agrees very well with a “lying-down” orientation of the adsorbed Creutz-Taube ions on the SiO_2_ surface with the charge compensation provided by the surface.

Besides the coverage results obtained from the XPS studies shown above, another possible way to get such orientation information is to measure the height or thickness of the Creutz-Taube ion monolayer. Different orientations of the Creutz-Taube ion on a surface would give a different thickness of the monolayer. So by measuring the height of the Creutz-Taube ion monolayer on surface, we should be able to get the orientation information. Ellipsometry is a useful tool to measure the thickness of surface films over a large area. We used ellipsometry to characterize the surface attachment of the Creutz-Taube ion on SiO_2_. Table 3 gives the thickness of the native oxide and the Creutz-Taube ion monolayer. The soaking time was kept longer than three hours in order to ensure a fully formed monolayer.

**Figure 10.**
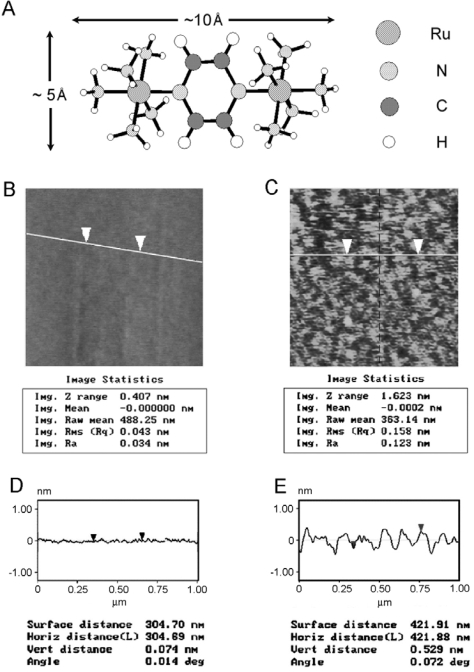
(A) Schematic structure of the Creutz-Taube ion with its lateral dimension illustrated. (B, C) AFM images of the SiO_2_ substrate before and after attachment of the Creutz-Taube ions. Increased RMS surface roughness (0.043 nm before attachment and 0.158 nm after attachment) is observed. Line-section analysis lines are also shown on the graphs. (C, D) Line-section analysis results of (B, C).

The ellipsometric thickness of the final Creutz-Taube ion monolayer is 6.4±0.4 Å. From Figure 10A, we know that if the Creutz-Taube ion stands up on the surface, we would expect a thickness of 10 Å of the resulting Creutz-Taube ion monolayer, whereas if the lying-down orientation is favored, a thickness of 5 Å would be obtained. Here the value of 6.4 Å indicates the adsorbed Creutz-Taube ion favors a lying-down orientation or at least this orientation dominates on the surface over the standing-up orientation if both orientations are mixed on the surface. However, for such mixed orientations, the surface roughness would be higher than the surface roughness when only one orientation were adopted on the surface.

In order to characterize the surface structures of the Creutz-Taube ion on silica at a microscopic level, atomic force microscope (AFM) was used. For line-section analysis, a partially covered Creutz-Taube ion film was prepared by soaking the SiO_2_ substrate in the solution for less than 30 min. Figures 10B and 10C show the corresponding AFM pictures of the SiO_2_ substrate before and after the attachment of the Creutz-Taube ions. In Figure 10C, we interpret the bright areas as patches of the Creutz-Taube ion covered layer and the dark areas as the uncovered SiO_2_ substrate. The coverage obtained from the AFM image is around 70 ∼ 80 %. From Figure 10E, we see the height difference between the Creutz-Taube ion domains and the substrate is about 5.3 Å, a value very close to the Creutz-Taube monolayer with a lying-down orientation. The surface roughness obtained on selected bright (the Creutz-Taube ion region) area is about 1.6 Å, which suggests a single orientation was adopted for the Creutz-Taube ion when bound to surface (to form a smooth monolayer). A complementary tool to obtain the height information of the covered area is height analysis, a statistical analysis tool from the AFM software that can give the height distributions for the island domains on the substrate. Here we obtained an average value of 0.515 ± 0.107 nm for these small islands, which also agrees well with the lying-down orientation of the attached Creutz-Taube ions on surface.

### 3.4. Nanolines of the Creutz-Taube Ions Patterned through High-Resolution PMMA Masks

**Figure 11.**
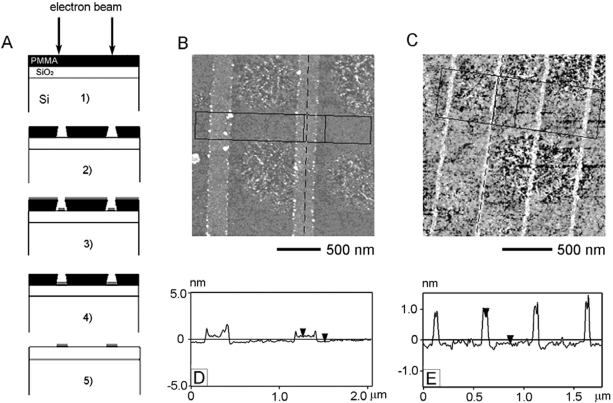
(A) Schematic illustration of the processes for nanopatterning of the Creutz-Taube ions on SiO_2_ substrate. (B, C) Tapping mode AFM images and (D,E) the corresponding line-section analysis of two 200 nm and 35 nm lines of the nanopatterened Creutz-Taube ions on SiO_2_.

Because of the high selective binding property of the Creutz-Taube ion on SiO_2_ and PMMA surface, a procedure of molecular nanopatterning of the Creutz-Taube ion is illustrated in Figure 11A. First a PMMA film is applied to a silicon dioxide layer on a Si substrate, and then an electron beam is used to draw patterns in the PMMA film. After development (to remove the exposed PMMA area in a developer solution), trenches are formed in the PMMA film. The following steps involve soaking the nanopatterned substrate in the Creutz-Taube ion solution and then followed by washing off the PMMA using either acetone or DCM.

Figures 11B and 11C show tapping mode AFM images of two samples prepared by this masking process. In Figure 11B, 200 nm wide lines (PMMA exposure dose 4.2nC/cm) of the patterned Creutz-Taube ions on SiO_2_ were demonstrated after the PMMA was removed by DCM. Figure 11D gives the average cross sectional profile confined by the box as indicated in Figure 11B. We can see that the height of the 200 nm wide Creutz-Taube ion lines is about 0.553 nm away from the edge of the Creutz-Taube ion line. Several segments of these two Creutz-Taube ion lines were measured, giving the height of the Creutz-Taube ion lines as from 4 to 6 Å. This result further confirms our conclusion on the monolayer formation of the Creutz-Taube ions on SiO_2_ in that if the Creutz-Taube ion layer is thicker than a monolayer, the height of the 200 nm wide line could vary considerably when different segments were measured, whereas a monolayer can be only one thickness, as observed here. From Figures 11B and 11D it is clear that the accumulated particles on the edges of the Creutz-Taube ion lines severely affect the measurement results of heights of the Creutz-Taube ion lines. The reason for the aggregation of material at the edges of the lines could be due to the formation of the high edged PMMA debris and is still under investigation. Figure 11C shows another example where a narrow Creutz-Taube ion lines (exposure dose 1.6 nC/cm, line width 35 nm) were patterned on SiO_2_ substrate after removal of the PMMA with DCM. Figure 11E gives the average cross-sectional profiles as shown in Figure 11C, here we found the height of the Creutz-Taube ion line is about 0.9 nm, a value higher than the expected thickness of 0.5 nm for the formed Creutz-Taube ion monolayer with a lying-down orientation. The reason for such an observation could be due to the effects of the high edged trenches and the convolution of the AFM tip. Since the width of these lines is around 35 nm, due to the formation of the high edged PMMA debris, the AFM tip (with a70 radius ∼ 15–30 nm) may not be able to get deep inside these trenches. So the height from such measurement may appear higher than expected. This effect can be eliminated for wider lines of the Creutz-Taube ions (as shown in Figure 11C) on the surface. Given the size of the Creutz-Taube ions (∼ 5 × 5 × 10 Å), it seems that the Creutz-Taube ions have been deposited preferentially in the exposed lines, with little or no diffusion to bare SiO_2_ occurring after removal of the remaining PMMA.

## 4. Conclusions

With the use of XPS, we have found that the Creutz-Taube ion can selectively bind only on hydrophilic types of surfaces, such as SiO_2_ and –OH terminated organic SAMs on gold, but not to hydrophobic surfaces, such as –CH_3_ terminated organic SAMs and poly(methylmethacrylate) (PMMA) thin films. The counter ions of the Creutz-Taube ions do not bind to the anionic SiO_2_ surfaces or onto any of the non-polar surfaces, although it binds to H-bonding HUT surfaces. Further ellipsometric, atomic force microscopy (AFM) and time-dependent XPS studies suggested that the attached cations could form an inorganic analog of the self-assembled monolayer on SiO_2_ substrate with a “lying-down” orientation. The strong electrostatic interaction between the highly charged cations and the anionic SiO_2_ surface was believed to account for these observations. Based on its selective binding property, patterning of wide (∼200 nm) and narrow (∼35 nm) lines of the Creutz-Taube ions on SiO_2_ surface were demonstrated through PMMA electron resist masks written by electron beam lithography (EBL).

## Figures and Tables

**Table 1.** Binding energy and the relative concentrations of *Ru*, *N* and *S* in the Creutz-Taube ion powder sample with toluenesulfonate (TOS) as the counter ions.

	Ru 3p	N 1s	S 2p
**Binding Energy (eV)**	461.8 (3p^3/2^) 484.7(3p^1/2^)	399.7	167.9
**Rel. Conc. (%)**	10.2	62.3	27.5

**Table 2.** Selective binding properties of the Creutz-Taube ions (CT5) on hydrophilic and hydrophobic substrates.

		Ru	N	S	P	F
**CT5(TOS)_5_ on SiO_2_**	B. E. (eV)	462.4 (3p^3/2^) 485.7 (3p^1/2^)	400.0 (1s)	No binding	--	--
	Rel. Conc. (/Ru)	1	5.78			
**CT5(TOS)_5_ on OTS/SiO_2_**	B. E. (eV)	No binding	No binding	No Binding	--	--
	Rel. Conc. (/Ru)					
**CT5(TOS)_5_ on PMMA/SiO_2_**	B. E. (eV)	No binding	No binding	No Binding	--	--
	Rel. Conc. (/Ru)					
**CT5(TOS)_5_ on HUT/Au**	B. E. (eV)	462.4 (3p^3/2^) 484.9 (3p^1/2^)	399.8 (1s)	168.1 (2p TOS) 162.0 (2p Thiol)	--	--
	Rel. Conc. (/Ru)	1	5.41	1.70 (2p TOS) 1.66 (2p Thiol)		
**CT5(TOS)_5_ on ODT/Au**	B. E. (eV)	No binding	No binding	No binding	--	--
	Rel. Conc. (/Ru)					
**CT5(PF_6_)_5_ Powder**	B. E. (eV)	462.7 (3p^3/2^) 484.5 (3p^1/2^)	399.7 (1s)	--	135.9 (2p)	686.5 (1s)
	Rel. Conc. (/Ru)	1	6.08		2.42	14.8
**CT5(PF_6_)_5_on SiO_2_**	B. E. (eV)	461.5 (3p^3/2^) 484.5 (3p^1/2^)	399.8 (1s)	--	No binding	Bo binding
	Rel. Conc. (/Ru)	1	6.40			

**Table 3.** Ellipsometric measurement of the Creutz-Taube ion monolayer on SiO_2_.

Native SiO_2_ thickness before attachment of CT5 (Å)	11.3	11.8	11.4	11.3	11.6	11.4	11.6	Average 11.5±0.2
Total Native SiO_2_ and CT5 thickness after attachment (Å)	18.0	18.6	17.6	17.9	17.8	18.1	17.6	Average 17.9±0.5
Calculated CT5 monolayer thickness (Å)	6.7	6.8	6.2	6.6	6.2	6.7	6.0	Average 6.4±0.4
